# Effects of Cadmium on Liver Function and Its Metabolomics Profile in the Guizhou Black Goat

**DOI:** 10.3390/metabo13020268

**Published:** 2023-02-13

**Authors:** Yuanfeng Li, Xiaoyun Shen

**Affiliations:** 1 College of Life Science and Engineering, Southwest University of Science and Technology, Mianyang 621010, China; 2 State Key Laboratory of Sheep Genetic Improvement and Healthy Production, Xinjiang Academy of Agricultural and Reclamation Sciences, Shihezi 832000, China

**Keywords:** metabolomics, cadmium, goat, liver

## Abstract

Cadmium (Cd) is a toxic heavy metal, which will lead to ecosystem contamination, threatening the life of grazing animals. Goats are an important grazing animal biomarker to evaluate Cd toxicity, but the effect of short-term and high-concentration Cd toxicity on goat liver function and its latent mechanism is still unclear. A total of ten male Guizhou black goats were randomly divided into two groups: CON group, sterilized tap water (no CdCl_2_), and Cd group (20 mg Cd·kg^−1^·BW, CdCl_2_⋅2.5H_2_O). The test lasted for 30 days. In this study, we found that Cd poisoning in drinking water affected significantly the distribution of Cd in the goat offal and tissues, and damaged the goat’s immune function of the liver. With a metabolomics approach, 59 metabolites were identified. Metabolomics analysis suggested that Cd affected lipid and amino acid metabolism of the goat liver. Collectively, our results confirmed the effect of Cd on liver function and liver metabolism, and provided insights on the molecular basis for early warnings of Cd poisoning in goats.

## 1. Introduction

Cadmium (Cd) is a heavy metal element with long biological half-life, slow metabolism, and strong toxicity, which easily accumulates in the body. Cd can cause damages to a variety of tissues and organs [1], thereby seriously threatening human health. With the extensive use of Cd in industrial production, environmental Cd pollution is becoming more and more serious. Cd and other heavy metals migrate and circulate in the ecosystem in various ways, and are accumulated in animals through different intake pathways, which will eventually lead to animal death or affect food safety. Long-term Cd exposure may directly cause diseases of the immune, respiratory, digestive, nervous, and reproductive systems [2]. Poisoning caused by long-term chronic accumulation of Cd has attracted more and more social attention, but the pathogenesis of short-term toxicity of high-dose Cd is not completely clear. 

Omics techniques, a high-throughput method, has been popularly applied in the process of disease diagnosis [3]. Metabolomics has been proved to be conducive to toxicological mechanism study, disease diagnosis, and treatment assessment [4]. Metabolomics also offers an opportunity to discover biomarkers for premalignant liver disease, thereby alerting the potential risk of impending hepatocellular carcinoma [5]. Metabolomics analyzes the changes of the total metabolites of biological samples affected by the environment. This helps to understand how animals and plants respond to various hostile factors at the molecular level [6]. At present, it is widely used in the study of environmental pollutants, such as investigating the mechanism of Cd poisoning in rat urine [7] and kidneys [6], which provides the possibility for directly evaluating the health effects of low-dose environmental poisons [8]. 

The purpose of this experiment was to explore whether oral Cd affected liver function, thereby indirectly interfering with liver metabolism. Screening of metabolic markers may provide a new method for the early diagnosis of Cd poisoning.

## 2. Animals, Materials and Methods 

### 2.1. Reagents and Chemicals 

Cadmium chloride (CdCl_2_) was purchased from Tianjinzhiyuan Chemical Reagen Co., Ltd. Formic acid (FC, MS grade) and acetonitrile (CAN, MS grade) were purchased from TCI (Shanghai, China) and Merck (Rahway, NJ, USA), respectively. 

### 2.2. Animal Handing and Sample Collection

A total of ten male Guizhou black goats, 10.72 ± 0.35 kg, were purchased from the goat breeding farm in Guizhou Academy of Agricultural Sciences, Guiyang, China. The goats were maintained in a sheepfold with wooden leak floor, and were provided with a standard diet [9] (CP 14.75%, ME 9.80 MJ/kg, NDF 20.40%) and sterilized tap water (Zn 0.52 mg/L, Cu 0.52 mg/L, Fe 0.11 mg/L, pH 6.70) freely. All the animals in the Cd group were provided with drinking water containing Cd (20 mg Cd·kg^−1^·BW, CdCl_2_⋅2.5H_2_O) according to the recommended dose of previous studies [10] and investigation results of our team [11], and the experimental diets were simultaneously provided.

CdCl_2_ was prepared in 5.7% aqueous solution, and each goat drank 1000 mL per day (containing 5 mL CdCl_2_ aqueous solution). Goats in the control group were treated with identical quantity of disinfected tap water, so as to clear up the impacts of CdCl_2_. After 1-week adaptation, all the goats were fed tap water or water added with CdCl_2_ for 30 days, then were fixed. On day 1 and day 30, the blood samples were collected into vacuum tubes with heparin sodium and were centrifuged at 3000× *g* for 10 min (4 °C), then were cryopreserved at −80 °C for mineral elements and omics analysis. After the tested animals were slaughtered using an electric shock gun (ASS-1, Jarvis Machinery Manufacturing Co., Ltd., Beijing, China), the organs and tissues of (heart, liver, spleen, etc.) were cryopreserved at −80 °C for further analysis. The liver tissues were fixed with 4% paraformaldehyde and then were embedded in paraffin to make a 5 μm–thick section for hematoxylin-eosin (HE) staining. An optical microscope was used to observe histopathological changes. Preparation and determination of liver pathological sections (HE staining) was tested by Wuhan Servicebio Biological Co., Ltd., Wuhan, China. 

The levels of minerals (Zn, Cu, iron (Fe), and Cd), were measured using a graphite furnace atomic absorption spectrophotometer (AA–7000, Shimadzu Corporation, Kyoto, Japan). Commercial test kits, purchased from Nanjing Jiancheng Bio-Engineering Institute, China, were used to analyze the concentrations of immunoglobulin G (IgG), immunoglobulin M (IgM), immunoglobulin A (IgA), interleukin 6 (IL-6), interleukin-1β (IL-1β), and tumor necrosis factor-alpha (TNF-α) [9]. 

### 2.3. Metabolomics Analysis in the Liver

#### 2.3.1. Liver Preparation for Metabolomics 

Liver samples were processed in the same manner as described by Li et al. [12]. A total of 2μL supernatant was leached through a 0.22 μm nylon filter and was injected for HILIC UHPLC-Q-EXACTIVE/MS analysis. The stability of the LC-MS/MS system was tested with five quality control samples in the analysis sequence, prepared from the pooled liver samples.

#### 2.3.2. Metabolomics Data Capture

An ultraperformance liquid chromatography system was used to carry out chromatographic separation of the liver (Ultimate 3000, Thermo Fisher, Waltham, MA, USA). The liver was injected on an ACQUITY UPLC BEH C18 column (2.1 × 100 mm, 1.7 μm) at 40 °C (0.30 mL/min flow rate). The optimal linear gradient program referred to the description of Shen et al. [13], and the optimal mobile phase included 0.1% formic acid in water (A) and acetonitrile (B).

HILIC UHPLC-Q-Exactive/MS (Thermo Fisher, Greenville, NC, USA), fitted with a dual electrospray ionization source (ESI) operating in positive and negative ion modes, was used to carry out mass data acquisition. The scan time was set at 5 spectra/s and the centroid mode was from 50 to 1000 m/z. The optimal conditions of analysis referred to the description of Shen et al. [13]. 

#### 2.3.3. Multivariate Statistical Analysis 

A Compound Discoverer 3.0 (Thermo Fisher, USA) was used to convert the raw MS spectra to common data format (.mzML). Candidate metabolites (VIP > 1 and adjusted *p* value < 0.05) were regarded as potential biomarkers. The supporting files provided further details regarding metabolomic analysis.

#### 2.3.4. Metabolites Identification and Pathway Analysis 

The metabolite structure was confirmed through LS-MS/MS analysis. The basis of the METLIN were used to search the accurate mass values of the metabolites and MS/MS fragment ions. The KEGG and HMDB database were used to search metabolic pathways and biochemical reactions. Pathway analysis and visualization were performed on Compound Discoverer 3.0 (Thermo Fisher Scientific) software. 

### 2.4. Statistical Analysis

Data were analyzed using independent sample *t*-test of one-way ANOVA program of SPSS software (version 23.0, Inc., Chicago, IL, USA), and were expressed as the mean. The fixed factor was the concentration of Cd, and the dependent factors were the contents of minerals in organs and tissues, and the levels of immune factors in serums and livers. A *p* value < 0.05 was considered statistically different.

## 3. Results 

### 3.1. The Effect of Cd on the Pathological Structure in the Liver of the Guizhou Black Goat 

**Figure 1:**
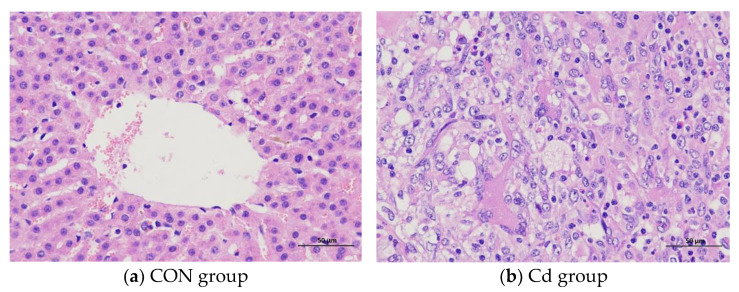
Histopathological changes of the liver in the Guizhou black goat (HE × 400).

The liver pathological structure under a light microscope showed that the hepatocytes of the black goat in the control group were closely arranged together with clear cells and outlines and clearly visible liver lobules. Hepatocyte cords were radially arranged around the central vein, and the trend was relatively clear (Figure 1a). Sheet necrosis could be seen in the hepatocytes of the black goat in the Cd group, with light and no staining in the nuclei, unclear boundaries of liver lobules, and disordered arrangement of hepatocyte cords (Figure 1b).

### 3.2. The Analysis of Mineral Contents in the Organ and Tissue

**Figure 2:**
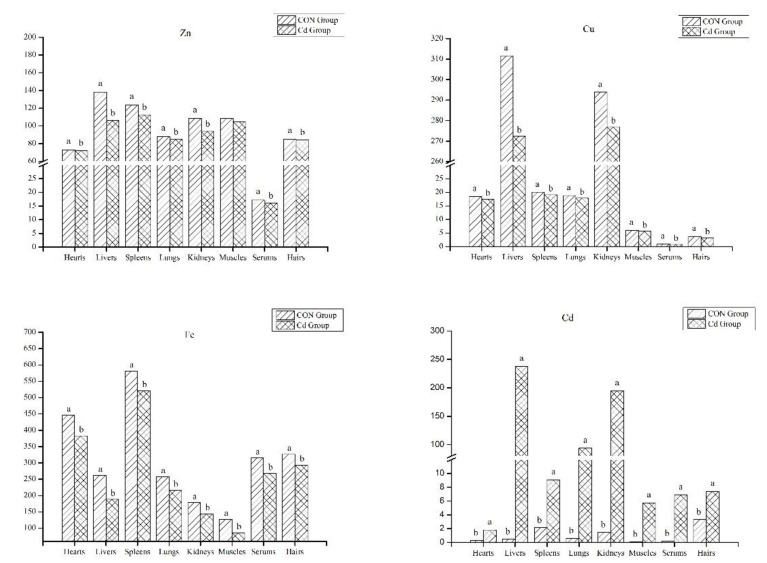
Mineral elements in the goat organ and tissue (ug/g). Zn, zinc; Cu, copper; Fe, iron; Cd, cadmium. Different small letters of superscript indicate significant differences (*p* < 0.05), and the same small letters or no letters indicate no significant difference (*p* > 0.05).

Metal contaminants can effectively be bioaccumulated by animals from the environment. The supplementation of Cd in drinking water significantly increased the Cd content in the organs (heart, liver, spleen, lung, kidney, muscle), serum, and hair of the Guizhou black goat (heart 1.82 vs. 0.31 ug/g, liver 237.94 vs. 0.46 ug/g, spleen 9.06 vs. 2.12 ug/g, lung 93.77 vs. 0.60 ug/g, kidney 194.65 vs. 1.44 ug/g, muscle 5.74 vs. 0.11 ug/g, serum 6.88 vs. 0.22 ug/mL, hair 7.41 vs. 3.32 ug/g, respectively) (*p* < 0.05, Figure 2), and greatly decreased the Cu and Fe levels compared with the control group (*p* < 0.05, Figure 2). The Cu levels in the heart, liver, spleen, lung, kidney, muscle, serum, and hair between the Cd group and the control group were 17.54 vs. 18.50 ug/g, 272.36 vs. 311.60 ug/g, 19.12 vs. 20.08 ug/g, 17.86 vs. 18.82 ug/g, 276.93 vs. 293.86 ug/g, 5.70 vs. 6.01 ug/g, 0.86 vs. 0.97 ug/mL, 3.20 vs. 3.80 ug/g, respectively (Figure 2). The Fe level in the heart, liver, spleen, lung, kidney, muscle, serum, and hair between the Cd group and the control group was 382.79 vs. 447.13 ug/g, 188.36 vs. 262.21 ug/g, 520.93 vs. 580.82 ug/g, 216.22 vs. 257.90 ug/g, 143.97 vs. 178.24 ug/g, 85.80 vs. 127.57 ug/g, 267.30 vs. 315.17 ug/mL, 293.67 vs. 327.12 ug/g, respectively (Figure 2). Compared with the control group, the Zn content in the heart, liver, spleen, lung, kidney, serum, and hair of the Guizhou black goat was significantly increased (72.20 vs. 73.08 ug/g, 105.97 vs. 138.32 ug/g, 112.56 vs. 123.62 ug/g, 84.70 vs. 87.86 ug/g, 93.63 vs. 108.39 ug/g, 104.81 vs. 108.64 ug/g, 15.99 vs. 17.19 ug/mL, 84.16 vs. 85.23 ug/g, respectively) (*p* < 0.05, Figure 2). Goats from the Cd group exhibited significant Cd accumulation in the offal. 

### 3.3. The Effect of Cd on the Immune Function of the Goat Serum and Liver

**Figure 3:**
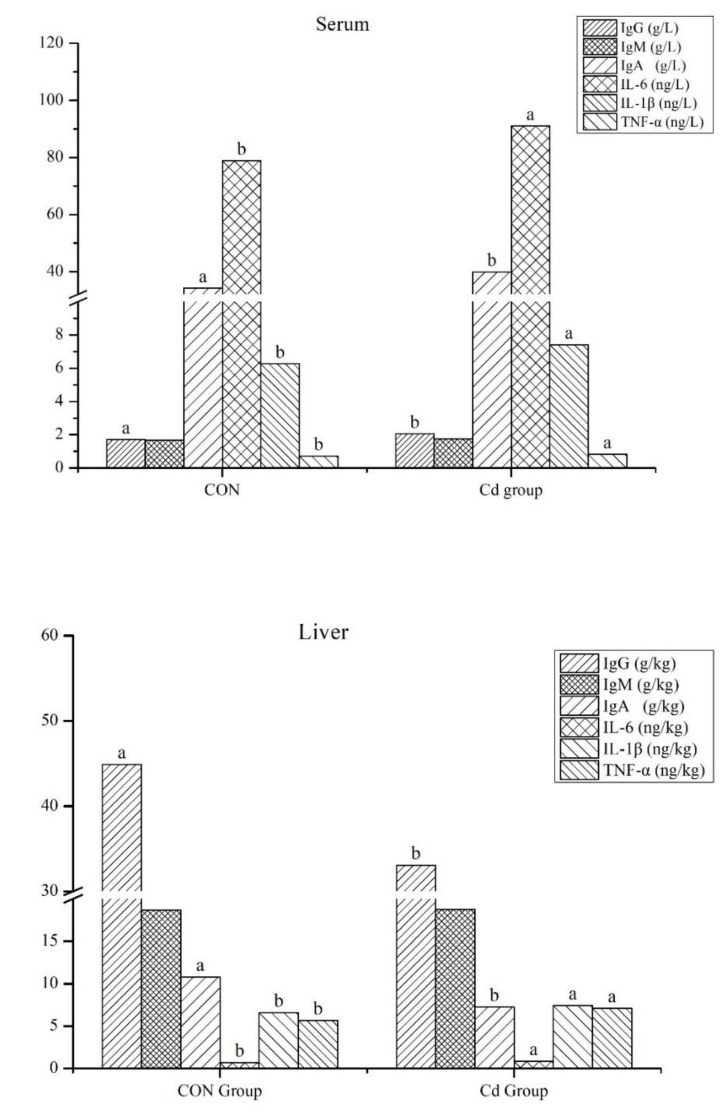
Immune functions in the goat serum and liver. IgG, Immunoglobulin G; IgM, Immunoglobulin M; IgA, Immunoglobulin A; IL-6, interleukin 6; IL-1β, interleukin-1β; TNF-α, tumor necrosis factor-alpha. CON Group, the control group; Cd Group, the cadmium group. Different small letters of superscript indicate significant differences (*p* < 0.05), and the same small letters or no letters indicate no significant difference (*p* > 0.05).

Compared with the control group, the levels of serum IgG and IgA in the Cd group were significantly decreased (2.05 vs. 1.70 g/L, 34.56 vs. 33.56 g/L, respectively), and the levels of serum IL-6, IL-1β, and TNF-α were significantly increased (78.82 vs. 91.05 ng/L, 6.27 vs. 7.41 ng/L, 0.72 vs. 0.83 ng/L, respectively) (*p* < 0.05, Figure 3). Compared with the control group, the levels of liver IgG and IgA in the Cd group were significantly decreased (44.90 vs. 33.10 g/kg, 10.80 vs. 7.23 g/kg, respectively), and the levels of liver IL-6, IL-1β, and TNF-α were significantly increased (0.69 vs. 0.84 ng/kg, 6.59 vs. 7.42 ng/kg, 5.66 vs. 7.09 ng/kg, respectively) (*p* < 0.05, Figure 3).

### 3.4. Liver Metabolic Responses in the Goat to Cd Exposure

**Figure 4:**
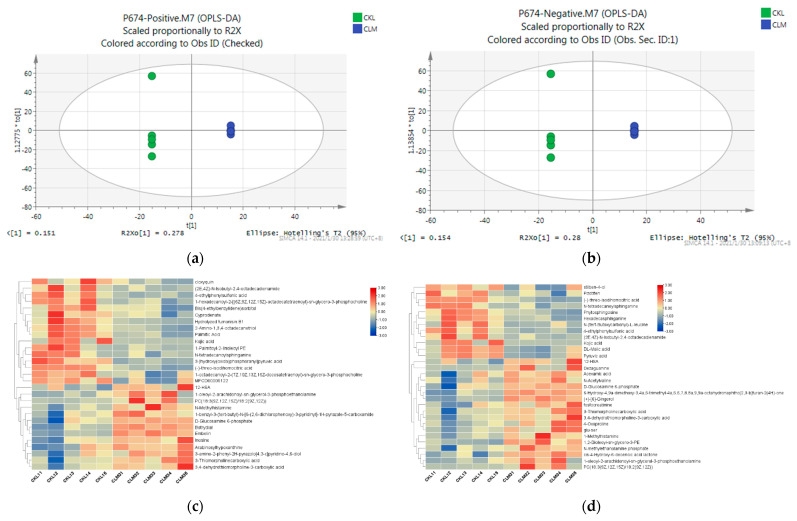
OPLS−DA score plots derived from HILIC UHPLC−Q−EXACTIVE/MS in positive (**a**) and negative (**b**) ionization modes. Samples in the Cd group were separated from those in the control group. R2 X and R2 Y indicate the fraction of the variables explained by the model, and the Q2 shows the predictive abilities of the model. Control versus Cd, ESI+: R2 X = 0.781, R2 Y = 1.000 and Q2 = 0.794; ESI-: R2 X = 0.799, R2 Y = 1.000 and Q2 = 0.802. Hierarchical clustering heat map, correction analysis, and pathway analysis of metabolites that were obtained from the liver. Unsupervised hierarchical clustering heat map of metabolites from liver of goats in the positive mode (**c**) and negative mode (**d**). Cluster of the changed metabolites (right side) and the samples in different groups (bottom). The color of the heatmap from red to blue indicated the relative intensity of the metabolites.

The typical total ion chromatograms of liver samples through the HILIC UHPLC−Q−EXACTIVE/MS analysis displayed good separation, peak shape, and strong intensity. Clear separations were displayed between the control and Cd groups in the well-fitting OPLS−DA models (Figure 4a,b). According to the VIP value (>1) and *p*-value (<0.05), 59 differential metabolites, including 31 up−regulated metabolites and 28 down−regulated metabolites, were identified (Table 1). Liver samples in the Cd group were separately clustered from the control group in the dendrogram of hierarchical clustering (Figure 4c,d). These results confirmed that the levels of most metabolites were affected by Cd. 

**Figure 5:**
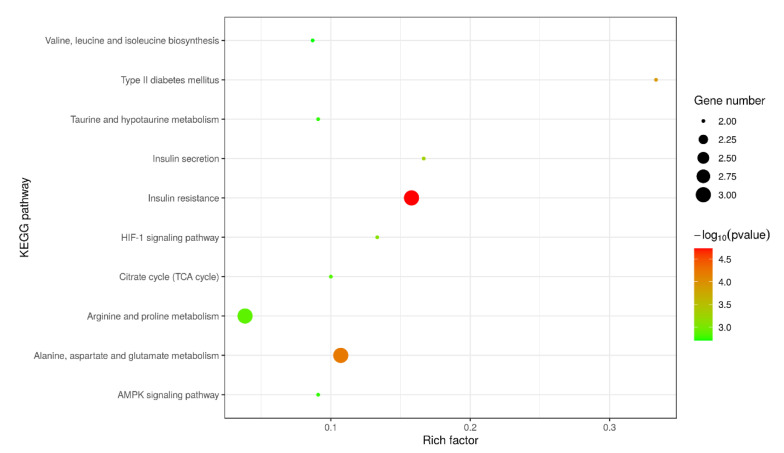
KEGG metabolic pathway analysis diagram of metabolites from livers of goats.

The liver differentially-expressed metabolites that were obtained in the treatment group were submitted to KEGG website for relevant pathway analysis. The most significant metabolic pathway was insulin resistance (Figure 5). Cd poisoning extremely altered metabolic pathways. The predominant metabolites were involved in insulin resistance, alanine, aspartate, and glutamate metabolism, arginine and proline metabolism, citrate cycle (TCA cycle), AMPK signaling pathway, etc. The details of related metabolic pathways are shown in Table 1. KEGG pathway analysis for the corresponding metabolic pathways found similar results that lipid metabolism was the main metabolic pathway (Table 2).

## 4. Discussion

### 4.1. Mineral Accumulation in the Goat Offal and Tissue and Liver Immune Function

In Cd-treated goat samples, severe Cd accumulation was observed in the Guizhou black goat. In the case of chronic Cd poisoning, about 8 % of Cd that is ingested will be absorbed by the body through the gastrointestinal tract [14]. In a previous study, Zalups [15] reported that rats were intravenously injected with 5 mol/kg Cd (source of CdCl_2_), and 50–60% of the administered dose of Cd was significantly accumulated in the liver. Cd is metabolized very slowly in the body, and its half-life is as long as 20~30 years. The human body can only excrete 0.005~0.015 % of the total amount of Cd in the body every day. In the case of chronic Cd poisoning, daily fecal Cd emission accounts for about 0.03 % of the total Cd in human body [16]. Long-term Cd accumulation will lead to oxidative stress, apoptosis or necrosis of generous cells, and even tumors [17]. In this trial, after high dose Cd was orally fed to the goats for 30 days, significant Cd accumulation occurred in the offal and tissue.

Antibodies and immunoglobulins that were produced by B cells, including IgG, IgM, and IgA, are important indicators of humoral immunity [18]. The synergy of immunoglobulin in the body can prevent body infection and resist the invasion of various bacteria and toxins. IgG plays a major role in immunity. After infection, IgM combines with complement to dissolve pathogens [19]. In local mucosal immunity, IgA mainly plays the role of an antibacterial, antiviral, and a “barrier” [20]. TNF-α is the most important inflammatory cytokine in the body’s stress response, and its biological effect can be expanded by inducing the synergistic effect of other inflammatory mediators [21]. IL-1β is a pro-inflammatory medium that mediates the inflammatory response [22]. IL-6 is a pro-inflammatory transmitter, which may aggravate the inflammatory reaction in the body and even lead to the deterioration of some diseases [23,24]. This study found that Cd poisoning decreased significantly the levels of IgG and IgA in the serum and liver, and increased significantly the levels of IL-6, IL-1β, and TNF-α in the serum and liver, which caused damage to the immune system of black goats. The results of liver immune functions further confirmed that Cd exposure damaged the immune function of goats and caused toxic damage to the liver.

### 4.2. Alteration of Energy and Lipid Metabolism Associated with Cd Exposures

The liver is the center of material storage and transformation. In mammals, glucose is preferentially used as an energy source, and the effect of fatty acid oxidation on energy supply is often related to the fat level in feed [25]. Under Cd poisoning, the energy metabolism state of black goats can be reshaped to adapt to a higher metabolic rate.

The tricarboxylic acid cycle (TCA cycle) is the link and transformation hub of sugar, fats, proteins, and even nucleic acid metabolism. Produced during aerobic oxidation of sugar, α-Ketoglutaric acid, pyruvic acid, and oxaloacetic acid can be converted into corresponding amino acids by combining with ammonia. The deamination of these amino acids can be converted into corresponding keto acids and enter the aerobic oxidation pathway of sugar. At the same time, glycerol that is produced by lipid catabolism and acetyl CoA that is produced by fatty acid metabolism can also enter the aerobic oxidation pathway of sugar for metabolism [26]. With the decrease of energy intake and the increase of consumption in the Cd poisoning stage of black goats, it is particularly important to study the physiological mechanism of metabolic disorders that are related to negative energy balance. Negative energy balance will cause changes in multiple metabolic pathways to maintain the physiological conditions that are required for growth [27].

Amino acids play an important role in the energy production of the TCA cycle [28]. After deamination, they can be converted into fat through gluconeogenesis, indicating that Cd-induced lipid metabolism disorder in goats has changed the metabolites that are related to lipid metabolism (D-glucosamine 6-phosphate and pyruvate). The up-regulation of D-glucosamine 6-phosphate and down-regulation of pyruvate in the Cd group showed that amino acid metabolism was related to lipid metabolism. Since pyruvate played a key role in energy, amino acid, and lipid metabolism, down-regulated pyruvate indicated that there was also interference in lipid synthesis/degradation [29]. In the KEGG enrichment analysis of different metabolites of Cd poisoning in this chapter, many pathways related to lipid metabolism, such as the metabolism of arachidonic acid and linoleic acid, were also significantly enriched by NADPH, GPX, etc.

### 4.3. Alteration of Amino Acid Metabolism Associated with Cd Exposures

Previous studies have reported that amino acid metabolism of male rats exposed to Cd was disturbed [30]. Compared with the adolescent rats in the control group, the levels of these amino acids (glutamic acid, aspartic acid, etc.) in the anterior part of the hypothalamus in the Cd group were decreased, thus regulating various physiological functions including the endocrine system, and the content of glutamine in the basal part of the hypothalamus was increased [30]. However, Cd exposure can induce inflammation in animals by increasing the excessive accumulation of ROS [31,32] or the disorder of glucose metabolism in animals [33]. Valine, one of the eight essential amino acids for the human body, can promote normal growth of the body, repair tissues, regulate blood sugar, and provide necessary energy. Leucine and valine are branched-chain amino acids. Together with isoleucine and valine, they repair muscles, control blood sugar, decompose and convert into glucose faster, provide energy to body tissues quickly, promote energy metabolism (glucose uptake, mitochondrial biogenesis, and fatty acid oxidation), provide energy for protein synthesis, and inhibit protein degradation [34]. In our work, metabolomic analysis showed that the D-glucosamine 6-phosphate regulating the metabolism of alanine, aspartic acid, glutamic acid, cysteine, and methionine in Cd-treated goats increased significantly, while the pyruvate regulating the metabolism of alanine, aspartic acid, and glutamic acid decreased significantly. In this study, many differential metabolites were enriched in pathways that were related to amino acid metabolism, and the abundance of essential amino acids such as valine and L-leucine in the liver of Cd-poisoned black goats was significantly down-regulated or up-regulated. In addition, many amino acid metabolic pathways changed significantly, such as alanine, aspartic acid, and glutamic acid metabolism, arginine and proline metabolism, valine, leucine and isoleucine biosynthesis, histidine metabolism, glycine, serine and threonine metabolism, phenylalanine metabolism, cysteine and methionine metabolism, tyrosine metabolism, amino acid biosynthesis, etc. Therefore, Cd poisoning may affect the tricarboxylic acid cycle, gluconeogenesis/glycolysis, and amino acid metabolism of black goats.

## 5. Conclusions

This study focused on the effect of oral Cd on the liver function and liver metabolism in goats. Cd altered the contents of mineral elements in the goat’s organs and tissues, and reduced significantly liver immune functions. The metabolomic analysis showed that the differential metabolites were significantly enriched in the pathways related to lipid metabolism, such as linoleic acid metabolism and arachidonic acid metabolism. Embelin may be potential metabolic markers of Cd poisoning in the goat liver. In general, oral Cd interfered with liver metabolism by decreasing the liver immune function, causing liver damage.

## Figures and Tables

**Table 1:** Metabolic pathways of candidate biomarkers in the goat liver related to Cd exposure.

Pathway ID	Pathway Name	Total	Pop Hit	*p*-Value_Adjusted
oas04931	Insulin resistance	12	19	0.000834
oas00250	Alanine, aspartate, and glutamate metabolism	12	28	0.00139
oas04930	Type II diabetes mellitus	12	6	0.00179
oas04911	Insulin secretion	12	12	0.00583
oas04066	HIF-1 signaling pathway	12	15	0.00739
oas00330	Arginine and proline metabolism	12	78	0.00878
oas00020	Citrate cycle (TCA cycle)	12	20	0.00878
oas04152	AMPK signaling pathway	12	22	0.00878
oas00430	Taurine and hypotaurine metabolism	12	22	0.00878
oas00290	Valine, leucine, and isoleucine biosynthesis	12	23	0.00878
oas04922	Glucagon signaling pathway	12	26	0.0102
oas00770	Pantothenate and CoA biosynthesis	12	28	0.0106
oas00010	Glycolysis/Gluconeogenesis	12	31	0.0106
oas00620	Pyruvate metabolism	12	31	0.0106
oas00730	Thiamine metabolism	12	31	0.0106
oas00030	Pentose phosphate pathway	12	35	0.0127
oas05230	Central carbon metabolism in cancer	12	37	0.0133
oas00650	Butanoate metabolism	12	42	0.0161
oas00900	Terpenoid backbone biosynthesis	12	45	0.0175
oas00340	Histidine metabolism	12	47	0.018
oas00053	Ascorbate and aldarate metabolism	12	49	0.0185
oas00260	Glycine, serine, and threonine metabolism	12	50	0.0185
oas00564	Glycerophospholipid metabolism	12	52	0.0191
oas00760	Nicotinate and nicotinamide metabolism	12	55	0.0195
oas00040	Pentose and glucuronate interconversions	12	55	0.0195
oas00440	Phosphonate and phosphinate metabolism	12	56	0.0195
oas00360	Phenylalanine metabolism	12	60	0.0214
oas00630	Glyoxylate and dicarboxylate metabolism	12	62	0.0219
oas00270	Cysteine and methionine metabolism	12	63	0.0219
oas00350	Tyrosine metabolism	12	78	0.0318
oas05231	Choline metabolism in cancer	12	11	0.0465
oas01200	Carbon metabolism	12	114	0.0602
oas01230	Biosynthesis of amino acids	12	128	0.072
oas01210	2-Oxocarboxylic acid metabolism	12	134	0.0759
oas00565	Ether lipid metabolism	12	25	0.0893
oas00600	Sphingolipid metabolism	12	25	0.0893
oas00062	Fatty acid elongation	12	40	0.136
oas00071	Fatty acid degradation	12	50	0.164
oas00061	Fatty acid biosynthesis	12	58	0.183
oas01100	Metabolic pathways	12	2702	0.199
oas01040	Biosynthesis of unsaturated fatty acids	12	74	0.217
oas00230	Purine metabolism	12	95	0.265
oas00520	Amino sugar and nucleotide sugar metabolism	12	108	0.289
oas01212	Fatty acid metabolism	12	122	0.314
oas02010	ABC transporters ABC	12	137	0.338

**Table 2:** Significantly modulated metabolites in the goat liver with Cd poisoning based on OPLS-DA.

NO	Adduction	RT (min)	*m*/*z*	Formula	Metabolite	KEGG Pathway	VIP	Change	*p*-Value
1	[M + H]+	0.161	142.02649	C_6_H_6_O_4_	Kojic acid	-	1.73	↓	0.034
2	[M + H]+	6.872	405.3448	C_22_H_47_NO_5_	Hydrolyzed fumonisin B1	-	2.03	↓	0.007
3	[M + H]+	10.033	715.51426	C_39_H_74_NO_8_P	1-Palmitoyl-2-linoleoyl PE	-	1.66	↓	0.045
4	[M + H]+	5.051	202.0293	C_8_H_10_O_4_S	4-ethylphenylsulfonic acid	-	2.15	↓	0.003
5	[M + H]+	6.84	317.29251	C_18_H_39_NO_3_	2-Amino-1,3,4-octadecanetriol	-	2.16	↓	0.002
6	[M + H]+	10.02	753.52898	C_42_H_76_NO_8_P	1-hexadecanoyl-2-[(6Z,9Z,12Z,15Z)-octadecatetraenoyl]-sn-glycero-3-phosphocholine	-	1.91	↓	0.015
7	[M + H]+	10.023	837.62212	C_48_H_88_NO_8_P	1-octadecanoyl-2-(7Z,10Z,13Z,16Z-docosatetraenoyl)-sn-glycero-3-phosphocholine	-	1.68	↓	0.040
8	[M + H]+	0.114	151.98775	C_3_H_5_O_5_P	3-[hydroxyl (oxido) phosphoranyl]pyruvic acid	-	2.28	↓	0.001
9	[M + H]+	0.1	179.01454	C_9_H_6_ClNO	cloxyquin	-	1.79	↓	0.026
10	[M + H]+	6.802	273.26635	C_16_H_32_O_2_	Palmitic Acid	map01100, Metabolic pathways|map00062, Fatty acid elongation|map01212, Fatty acid metabolism|map00061, Fatty acid biosynthesis|map00071, Fatty acid degradation|map01040, Biosynthesis of unsaturated fatty acids	2.12	↓	0.004
11	[M + H]+	0.157	220.05786	C_8_H_12_O_7_	(-)-threo-isodihomocitric acid	-	2.35	↓	0.000
12	[M + H]+	10.156	511.49544	C_32_H_65_NO_3_	N-tetradecanoylsphinganine	-	1.78	↓	0.027
13	[M + H]+	7.502	414.20355	C_24_H_30_O_6_	Bis (4-ethylbenzylidene) sorbitol	-	1.77	↓	0.029
14	[M + H]+	9.484	335.31808	C_22_H_41_NO	(2E,4Z)-N-Isobutyl-2,4-octadecadienamide	-	1.69	↓	0.039
15	[M + H]+	6.556	227.18827	C_13_H_25_NO_2_	Cyprodenate	-	1.65	↓	0.046
16	[M + H]+	7.953	196.08868	C_14_H_12_O	MFCD00005122	-	1.79	↓	0.026
17	[M + H]+	7.251	310.17798	C_17_H_26_O_5_	Botrydial	-	2.34	↑	0.000
18	[M + H]+	7.19	494.1179	C_26_H_24_Cl_2_N_4_O_2_	1-benzyl-3-(tert-butyl)-N-[6-(2,6-dichlorophenoxy)-3-pyridinyl]-1H-pyrazole-5-carboxamide	-	1.71	↑	0.037
19	[M + H]+	7.078	294.18313	C_17_H_26_O_4_	Embelin	-	2.44	↑	0.000
20	[M + H]+	1.004	268.08046	C_10_H_12_N_4_O_5_	Inosine	map01100, Metabolic pathways|map02010, ABC transporters|map00230, Purine metabolism	1.76	↑	0.030
21	[M + H]+	1.307	268.08064	C_10_H_12_N_4_O_5_	Arabinosylhypoxanthine	-	1.77	↑	0.028
22	[M + H]+	4.912	242.08048	C_12_H_10_N_4_O_2_	3-amino-2-phenyl-2H-pyrazolo [4,3-c]pyridine-4,6-diol	-	1.63	↑	0.049
23	[M + H]+	9.156	300.26627	C_18_H_36_O_3_	12-HSA	-	1.63	↑	0.050
24	[M + H]+	0.997	147.03538	C_5_H_9_NO_2_S	3-Thiomorpholinecarboxylic acid	-	1.81	↑	0.024
25	[M + H]+	0.943	259.04518	C_6_H_14_NO_8_P	D-Glucosamine 6-phosphate	map01100, Metabolic pathways|map00520, Amino sugar and nucleotide sugar metabolism|map00250, Alanine, aspartate and glutamate metabolism|map04931, Insulin resistance	1.84	↑	0.021
26	[M + H]+	0.833	125.09551	C_6_H_11_N_3_	N-Methylhistamine	map01100, Metabolic pathways|map00340, Histidine metabolism	1.83	↑	0.021
27	[M + H]+	1.012	145.01971	C_5_H_7_NO_2_S	3,4-dehydrothiomorpholine-3-carboxylic acid	-	1.72	↑	0.035
28	[M + H]+	10.5	765.5307	C_43_H_76_NO_8_P	1-oleoyl-2-arachidonoyl-sn-glycerol-3-phosphoethanolamine	-	1.74	↑	0.033
29	[M + H]+	9.744	779.54458	C_44_H_78_NO_8_P	PC(18:3(9Z,12Z,15Z)/18:2(9Z,12Z))	-	1.64	↑	0.048
30	[M − H]-	0.175	142.02648	C_6_H_6_O_4_	Kojic acid	-	1.70	↓	0.035
31	[M − H]-	5.08	202.0293	C_8_H_10_O_4_S	4-ethylphenylsulfonic acid	-	2.12	↓	0.003
32	[M − H]-	1.082	231.14672	C_11_H_21_NO_4_	N-(tert-Butoxycarbonyl)-L-leucine	-	1.70	↓	0.035
33	[M − H]-	6.882	317.2925	C_18_H_39_NO_3_	Phytosphingosine	map00600, Sphingolipid metabolism|map01100, Metabolic pathways	2.12	↓	0.003
34	[M − H]-	6.844	273.26635	C_16_H_35_NO_2_	Hexadecasphinganine	-	2.08	↓	0.004
35	[M − H]-	0.145	220.05786	C_8_H_12_O_7_	(-)-threo-isodihomocitric acid	-	2.30	↓	0.000
36	[M − H]-	10.195	511.49544	C_32_H_65_NO_3_	N-tetradecanoylsphinganine	-	1.78	↓	0.025
37	[M − H]-	1.006	134.0203	C_4_H_6_O_5_	DL-Malic acid	-	1.99	↓	0.008
38	[M − H]-	8.716	295.13857	C_19_H_21_NS	Pizotifen	-	1.66	↓	0.042
39	[M − H]-	1.001	88.01482	C_3_H_4_O_3_	Pyruvic acid	-	1.94	↓	0.011
40	[M − H]-	9.524	335.31808	C_22_H_41_NO	(2E,4Z)-N-Isobutyl-2,4-octadecadienamide	-	1.66	↓	0.041
41	[M − H]-	7.997	196.08868	C_14_H_12_O	stilben-4-ol	-	1.65	↓	0.044
42	[M − H]-	7.294	310.17798	C_17_H_26_O_5_	6-Hydroxy-4,9a-dimethoxy-3,4a,5-trimethyl-4a,5,6,7,8,8a,9,9a-octahydronaphtho[2,3-b]furan-2(4H)-one	-	2.33	↑	0.000
43	[M − H]-	7.421	196.14558	C_12_H_20_O_2_	cis-4-Hydroxy-6-decenoic acid lactone	-	2.22	↑	0.001
44	[M − H]-	7.121	294.18313	C_17_H_26_O_4_	(+)-[6]-Gingerol	-	2.43	↑	0.000
45	[M − H]-	0.905	193.02524	C_4_H_8_N_3_O_4_P	fosfocreatinine	-	1.63	↑	0.046
46	[M − H]-	0.937	155.03456	C_3_H_10_NO_4_P	N-methylethanolamine phosphate	map00564, Glycerophospholipid metabolism	1.68	↑	0.039
47	[M − H]-	4.526	173.10491	C_8_H_15_NO_3_	Acexamic acid	-	1.61	↑	0.049
48	[M − H]-	9.199	300.26627	C_18_H_36_O_3_	12-HSA	-	1.63	↑	0.046
49	[M − H]-	1.011	129.04142	C_5_H_7_NO_3_	4-Oxoproline	map01100, Metabolic pathways|map00330, Arginine and proline metabolism	1.68	↑	0.038
51	[M − H]-	0.956	259.04518	C_6_H_14_NO_8_P	D-Glucosamine 6-phosphate	map01100, Metabolic pathways|map00520, Amino sugar and nucleotide sugar metabolism|map00250, Alanine, aspartate and glutamate metabolism|map04931, Insulin resistance	1.79	↑	0.023
52	[M − H]-	3.594	159.08951	C_7_H_13_NO_3_	N-Acetylvaline	-	1.73	↑	0.031
53	[M − H]-	1.023	147.03538	C_5_H_9_NO_2_S	3-Thiomorpholinecarboxylic acid	-	1.82	↑	0.020
54	[M − H]-	0.857	125.09551	C_6_H_11_N_3_	1-Methylhistamine	map01100, Metabolic pathways|map00340, Histidine metabolism	1.77	↑	0.026
55	[M − H]-	1.04	145.01971	C_5_H_7_NO_2_S	3,4-dehydrothiomorpholine-3-carboxylic acid	-	1.71	↑	0.034
56	[M − H]-	10.536	765.53073	C_43_H_76_NO_8_P	1-oleoyl-2-arachidonoyl-sn-glycerol-3-phosphoethanolamine	-	1.70	↑	0.035
57	[M − H]-	10.028	743.54515	C_41_H_78_NO_8_P	1,2-Dioleoyl-sn-glycero-3-PE	-	2.26	↑	0.001
58	[M − H]-	0.985	234.08487	C_8_H_14_N_2_O_6_	glu-ser	-	1.78	↑	1.782
59	[M − H]-	0.159	150.05415	C_6_H_6_N_4_O	Dezaguanine	-	1.63	↑	1.633

RT: retention time. ↑ indicates up-regulation and ↓ indicates down-regulation.

## Data Availability

The datasets that were generated during and/or analyzed during the current study are available from the corresponding author on reasonable request. The data are not publicly available due to the school regulations.
